# The Hospital Annual
*Burdett's "Hospital Annual, 1889." Containing a review of the position and requirements of the voluntary charities, and an exhaustive record of hospital and nursing work for the year. London : *The Hospital* office, 139-40 Salisbury-court, Fleet-street, E.C. Price 2s. 6d.


**Published:** 1889-05-11

**Authors:** 


### THE HOSPITAL ANNUAL.*

THE publication of "The Hospital Annual" is a more important fact than the aspect of the modest red volume would imply. It represents a task hitherto unattempted in connection with our medical charities. Hitherto, every hospital has gone its own way with an independence that amounted to irresponsibility. A house committee might enquire into one detail of expenditure or another, but it was a wholly private matter, and their action was very rarely guided by any practical knowledge of any hospital but their own. They had no opportunity of judging how much it cost to other institutions of similar size, and engaged in similar work. No individual man has time to go here and there asking how much is expended on salaries, provisions, etc., and, moreover, no individual man has the right to do so. Private enquiries are usually evaded, more or less courteously, by secretaries and superintendents. Private curiosity is often regarded as impertinent; public curiosity never is; and the very officials who would resent Mr. A. or Mr. B. asking to see their accounts, responded readily in almost all cases to the invitation sent by the publisher of *The Hospital* to submit the statistics of the institution to him. The few institutions that did not comply with the request were for the most part private establishments, maintained for the benefit of one man, and, though no doubt useful in their way, were not what is generally understood by the word "hospital." It cannot be said that the "Annual" is perfect. Many of the reports sent in were models of clearness, fulness, and accuracy, but others were the reverse. From this lack of unity—more of comprehension than purpose—the "Annual" suffers; but, such as it is, it provides an amount of information regarding hospitals, their work, and the cost of working them, such as has never before been collected.

There are many important questions raised in the mind of anyone who peruses it. It is, of course, impossible for any statistics, however clear and accurate, to tell the absolute value of any institution. Extravagance does not mean efficiency; too often expenditure is not justified by results; but one cannot help imagining that where salaries are very low the nursing and other service is not of the best. But the great bar to anything like true comprehension of the proportionate expenditure of a hospital is the out-patient. He does not cost much individually; he averages only a shilling a head, and a penny or two on either side will mark the extremes of his demand, but when he is one of thousands he forms an important item in hospital expenditure. If the cost of the out-patient department were deducted from the rest of the expenditure, it would be easier to form an approximate estimate of the extravagance or economy practised; but it is exceptional for this to be done, and in calculating the cost of each occupied bed we are often adding to it the cost of a considerable number of out-patients.

We have before referred to the extreme difficulty of forming a perfectly reliable comparison between hospitals; but we insist on it in the hope that it will impress on the secretaries of these institutions the necessity for full and detailed statements. Nevertheless, it is apparent from even a cursory glance at "The Hospital Annual" that provincial hospitals are managed at less cost than London ones, and Irish than Scotch. A closer scrutiny shows where the comparative economy is effected, and thereby gives one a fair idea of whether or not it affects efficiency. The notes given under the heading "Special Features" will often explain an apparently large expenditure. Exceptional length of residence in the wards, as at the National Hospital for the Paralysed and Epileptic, or the reception of cases requiring expensive treatment, as in the cancer ward of the Middlesex Hospital, often shows good cause for a sum per occupied bed that otherwise seems unduly large. From the proportion of beds constantly occupied to the available accommodation, we may gather if the hospital provides well or ill for the needs of the surrounding population; and by comparing the same with the amount of income, especially if it shows a deficit to the expenditure, we shall guess pretty fairly if the rich of the neighbourhood do their part towards supporting medical charities. We propose in a future article to deal with the statistics given in the "Hospital Annual"; but at present we do no more than indicate its scope and usefulness.

* Burdett's "Hospital Annual, 1889." Containing a review of the position and requirements of the voluntary charities, and an exhaustive record of hospital and nursing work for the year. London: *The Hospital* office, 139-40 Salisbury-court, Fleet-street, E.C. Price 2s. 6d.

